# Silymarin Protects Epidermal Keratinocytes from Ultraviolet Radiation-Induced Apoptosis and DNA Damage by Nucleotide Excision Repair Mechanism

**DOI:** 10.1371/journal.pone.0021410

**Published:** 2011-06-22

**Authors:** Santosh K. Katiyar, Sudheer K. Mantena, Syed M. Meeran

**Affiliations:** 1 Department of Dermatology, University of Alabama at Birmingham, Birmingham, Alabama, United States of America; 2 Comprehensive Cancer Center, University of Alabama at Birmingham, Birmingham, Alabama, United States of America; 3 Nutrition Obesity Research Center, University of Alabama at Birmingham, Birmingham, Alabama, United States of America; 4 Center for Aging, University of Alabama at Birmingham, Birmingham, Alabama, United States of America; 5 Birmingham Veterans Affairs Medical Center, Birmingham, Alabama, United States of America; Roswell Park Cancer Institute, United States of America

## Abstract

Solar ultraviolet (UV) radiation is a well recognized epidemiologic risk factor for melanoma and non-melanoma skin cancers. This observation has been linked to the accumulation of UVB radiation-induced DNA lesions in cells, and that finally lead to the development of skin cancers. Earlier, we have shown that topical treatment of skin with silymarin, a plant flavanoid from milk thistle (*Silybum marianum*), inhibits photocarcinogenesis in mice; however it is less understood whether chemopreventive effect of silymarin is mediated through the repair of DNA lesions in skin cells and that protect the cells from apoptosis. Here, we show that treatment of normal human epidermal keratinocytes (NHEK) with silymarin blocks UVB-induced apoptosis of NHEK *in vitro*. Silymarin reduces the amount of UVB radiation-induced DNA damage as demonstrated by reduced amounts of cyclobutane pyrimidine dimers (CPDs) and as measured by comet assay, and that ultimately may lead to reduced apoptosis of NHEK. The reduction of UV radiation-induced DNA damage by silymarin appears to be related with induction of nucleotide excision repair (NER) genes, because UV radiation-induced apoptosis was not blocked by silymarin in NER-deficient human fibroblasts. Cytostaining and dot-blot analysis revealed that silymarin repaired UV-induced CPDs in NER-proficient fibroblasts from a healthy individual but did not repair UV-induced CPD-positive cells in NER-deficient fibroblasts from patients suffering from xeroderma pigmentosum complementation-A disease. Similarly, immunohistochemical analysis revealed that silymarin did not reduce the number of UVB-induced sunburn/apoptotic cells in the skin of NER-deficient mice, but reduced the number of sunburn cells in their wild-type counterparts. Together, these results suggest that silymarin exert the capacity to reduce UV radiation-induced DNA damage and, thus, prevent the harmful effects of UV radiation on the genomic stability of epidermal cells.

## Introduction

Ultraviolet (UV) radiation is a well established etiologic risk factor for the incidence of melanoma and non-melanoma skin cancers, and these skin cancers are a major burden on the health care system. The incidence of skin cancers is equivalent to the incidence of malignancies in all other organs combined [1]. One of the hallmark events of exposure to UVB radiation (290–320 nm) is the induction of apoptotic cell death of keratinocytes, the results of which are evident within the epidermis as sunburn cells (SC) [2]. The formation of sunburn cells in UV-exposed skin indicates the severity of DNA damage. The repair of DNA damage in UVB-exposed skin cells can prevent the accumulation of damaged cells. If cells are not repaired, they may continue to replicate and may lead to cutaneous malignancies. This means that DNA repair process is a protective mechanism. Alternatively, induction of apoptosis of keratinocytes to UVB radiation is also a protective mechanism relevant in limiting the survival of cells with irreparable DNA damage. Changes in UV-induced apoptosis may therefore have significant impact on photocarcinogenesis. The molecular pathways leading to UVB radiation-induced apoptosis include the formation of cyclobutane pyrimidine dimers (CPDs) and (6–4) photoproducts [3], [4], the activation of death receptors including CD95 (Fas/APO-1) [3], [5], and the formation of reactive oxygen species [6]. These pathways are orchestrated by positive and negative factors that act within the epidermis in an autocrine and/or paracrine manner. Because of its protective function, alterations in UVB-induced apoptosis may have a profound impact in the induction of skin cancer, the most common cutaneous malignancy in Caucasians.

We have shown that topical application of silymarin, a plant flavanoid from milk thistle (*Silybum marianum)*, results in protective effects on UVB-induced skin carcinogenesis in mice [7]. Silymarin is composed primarily of silibinin (≈90%) together with small amounts of other silibinin stereoisomers, such as isosilybin, dihydrosilybin, silydianin and silychristin [7], [8]. It has been shown that silymarin inhibits photocarcinogenesis through inhibition of UVB-induced oxidative stress, inflammation and suppression of immune system, etc. [8]–[10]. However, precise mechanism of skin cancer chemoprevention by silymarin is still not very well understood. It is also unclear whether silymarin has the ability to decrease UV radiation-induced apoptotic cell death of epidermal cells through repair of damaged DNA and thus lead to prevention of photocarcinogenesis. Therefore, we hypothesized that prevention of photocarcinogenesis by silymarin is mediated through repair of UV-induced DNA damage in epidermal cells. To understand the precise mechanism, we have conducted series of experiments *in vitro* and *in vivo* and determined whether silymarin suppresses UV-induced apoptosis in skin cells and that this occurs through repair of damaged DNA. Here we report that treatment of normal human epidermal keratinocytes (NHEK) with silymarin inhibits UVB-induced apoptosis of keratinocytes, and in this process UVB-induced DNA damage was significantly reduced or repaired after silymarin treatment. Our study also reveals that the repair of UV-induced DNA damage or genomic instability by silymarin is mediated through the nucleotide excision repair (NER) mechanism, which was verified by using NER-proficient and NER-deficient human fibroblasts, and NER-deficient mouse model.

## Materials and Methods

### Chemicals and antibodies

Silymarin, agarose and other chemicals used in this study were of analytical grade and were purchased from Sigma Chemical Co. (St. Louis, MO). The primary antibody used against CPD was obtained from Kamiya Biomedical Company (Seattle, WA). The secondary antibodies, HRP-linked goat anti-mouse IgG was purchased from Santa Cruz Biotechnology, Inc. (Santa Cruz, CA). The Annexin V-conjugated AlexaFluor488 Apoptosis Detection Kit was purchased from Molecular Probes Inc. (Eugene, OR). Cell Death Detection ELISA^PLUS^ kit for cell apoptotic analysis was obtained from Roche-Applied Science (Mannheim, Germany). Keratinocyte growth medium supplemented with human recombinant epidermal growth factor and bovine pituitary extract was the product of Gibco/Invitrogen (Carlsbad, CA).

### Animals

The xeroderma pigmentosum complementation group A (*XPA*)-deficient mice, which are devoid of NER function, were generated and maintained in our animal resource facility as described previously [11], [12]. Female C3H/HeN mice (wild-type of *XPA*-deficient mice) of six to seven weeks old were purchased from Charles River Laboratory (Wilmington, MA). All mice were maintained under standard conditions of a 12-h dark/12-h light cycle, a temperature of 24±2°C, and relative humidity of 50±10%. The animal protocol used in this study was approved by the Institutional Animal Care and Use Committee of the University of Alabama at Birmingham, and approved Animal Protocol Number is: 100409095.

### Normal human epidermal keratinocytes, cell culture and treatment with silymarin

The NHEK were obtained from the Skin Diseases Research Centre core facility at the University of Alabama at Birmingham, AL. The primary skin cell cultures provided to researchers are derived from discarded, unidentified, remnant human skin tissue; therefore, use for research purposes is exempt from HIPAA regulation. Under this provision, informed consent from the human subjects is not required. These human skin tissues were collected from the Kirklin Clinic, University Hospitals of the University of Alabama at Birmingham, AL. The Institutional Review Board approval number is 040325001 for the UAB Skin Diseases Research Center, Core B: Skin Cell Culture Core. The NHEK were cultured in keratinocyte growth medium supplemented with 5 ng/ml human recombinant epidermal growth factor and 0.05 mg/ml bovine pituitary extract (Gibco/Invitrogen, Carlsbad, CA) and maintained in an incubator under standard cell culture conditions *viz.* temperature 37°C and 5% CO_2_ in humid environment, as detailed previously [13]. In all treatments, silymarin was dissolved initially in ethanol (final concentration 0.1% w/v) and made up to the required concentration with complete cell culture medium. The sub-confluent cells (60–70%) were treated with either varying concentrations of silymarin or vehicle alone (ethanol, 0.1% (v/v) in media) that served as a control. All the pre-treatments of cells with silymarin were done 3 h prior to the UVB exposure, otherwise stated.

### Xeroderma pigmentosum complementation Group A (XPA)-proficient and XPA-deficient human fibroblasts and fibroblast culture

*XPA*-deficient and *XPA*-proficient human fibroblasts were obtained from the Coriell Institute for Medical Research (Camden, NJ). The *XPA*-deficient fibroblasts were obtained from patients suffering from xeroderma pigmentosum group-A disease, while *XPA*-proficient fibroblasts were obtained from healthy individuals. These fibroblasts were authenticated by the Coriell Institute for Medical Research and supplied for only research purpose. The fibroblasts were cultured in Modified Eagle Medium with Earle's salts supplemented with 2 mM L-glutamine, 10% fetal bovine serum and maintained in an incubator at 37°C in a humidified atmosphere of 5% CO_2_, as detailed previously [14]. Cells were treated with silymarin as detailed above.

### UVB exposure of the cells

Cells were irradiated with UVB radiation (150 J/m^2^) through phosphate buffer saline (PBS). For this purpose cells were kept under UVB lamps (Daavlin, UVA/UVB Research Irradiation Unit, Bryan, OH) equipped with an electronic controller at the distance of 30 cm, and exposed them. Majority of the wavelengths of UV radiation were in the UVB (290–320 nm) range.

### Detection of cell death by ELISA

UV-induced cell death in NHEK or NER-deficient and NER-proficient cells was detected using Cell Death Detection ELISA^PLUS^ Kit (Mannheim, Germany). This kit was used to detect DNA fragmentation; mono- and oligonucleosomes released into the cytoplasm by biotinylated anti-histone- and peroxidase-coupled anti-DNA antibodies, following the manufacturer's protocol. Optical density was measured and is shown on the *y*-axis as mean±SD of triplicates.

### Quantification of apoptotic cells by FACS analysis

UVB-induced apoptosis in NHEK was determined by flow cytometry using the Annexin V-conjugated Alexafluor488 (Alexa488) Apoptosis Detection Kit following the manufacturer's instructions and as previously described by us [13]. Briefly, after overnight serum starvation, cells were treated with silymarin (0, 10 and 20 µg/mL) for 3 h then exposed to UVB (150 J/m^2^) through PBS. The cells were harvested 24 h later, washed in PBS and incubated with Alexa488 and propidium iodide for cellular staining in binding buffer at room temperature for 10 min in the dark, as detailed previously [15]. The stained cells were analyzed by FACS using a FACSCalibur instrument (BD Biosciences, San Jose, CA) equipped with CellQuest 3.3 software. The Alexa488-positive cells which have green fluorescence are termed as early apoptotic cells while propidium iodide-positive plus Alexa488-positive cells were termed as late apoptotic cells and had red-green fluorescence.

### Analysis of DNA damage by the Comet assay

UVB-induced DNA damage on per cell basis was determined using the comet assay, as described previously [15]. NHEK pre-treated with silymarin (0 or 20 µg/ml) for 3 h or non-silymarin-treated NHEK were exposed to UVB (200 J/m^2^), and were harvested 36 h later for comet assay. Briefly, after treatment with UVB the cells were harvested and re-suspended in ice cold PBS. Approximately, 1×10^4^ cells in a volume of 75 µL of 0.5% (w/v) low melting point agarose were pipetted onto a frosted glass slide coated with a thin layer of 1.0% (w/v) agarose, covered with a cover slip and allowed to set on ice for 10 min. Following removal of the cover slip the slides were immersed in ice-cold lysis solution containing 2.5 M NaCl, 10 mM Tris, 100 mM Na_2_-EDTA, 1% (w/v) N-lauroyl-sarcosine, adjusted to pH 10.0, and 1.0% Triton X-100 was added immediately before use. After 2 h at 4°C, the slides were placed into a horizontal electrophoresis tank filled with buffer (0.3 M NaOH, 1 mM EDTA (pH 13) and subjected to electrophoresis for 30 min at 300 mA. Slides were transferred to neutralization solution (0.4 M Tris-HCl) for 3×5 min washes and stained with ethidium bromide for 5 min. Slides were viewed using the 20x objective of a Zeiss Axioskop microscope equipped with epifluorescence optics. For each sample the tail lengths (µm) of a minimum of 30 comets were analyzed. The length of the comet was quantified as the distance from the centrum of the cell nucleus to the tip of the tail in pixel units and the tail length was expressed as a mean±SD from 30 comets.

### Immunohistochemical detection of CPD-positive cells

UV-induced DNA damage in the form of CPD^+^ cells were detected using a protocol described previously with some modifications [14]. Briefly, at indicated time point after UV irradiation (150 J/m^2^), cells were trypsinized and centrifuged. Cell pellets were resuspended in PBS buffer and processed for cytospin preparation (≈1×10^5^ cells/slide). Cells were washed in PBS and fixed with 45% ethanol for 5 min followed by 70% ethanol at −20°C for 10 min. Cells were subsequently permeabilized with 0.3% Triton×100 for 30 min. DNA denaturation was performed by treating the cells with 0.5 N HCl and 0.05% pepsin at 37°C for 30 min. Slides were then incubated with the CPD-specific monoclonal antibody for 1 h at room temperature and after washing the bound anti-CPD antibody was detected by incubation with biotinylated goat anti-mouse IgG1 followed by peroxidase labeled streptavidin. Cells were then incubated with diaminobenzidine plus peroxidase substrate for 5 min. After washing with distilled water, the cells were counterstained with harris haematoxylin. CPD^+^ cells were counted under Olympus BX41 microscope at 5–6 different fields and the data were presented as the mean of the percentage of CPD^+^ cells±SD from at least three separate experiments.

### Assay of CPDs by dot-blot analysis

Genomic DNA from the NHEK or XPA cells was isolated following the standard procedure, as described previously [11]. Genomic DNA (500 ng) was transferred to a positively-charged nitrocellulose membrane by vacuum dot-blotting (Bio-Dot Apparatus, Bio-Rad, Hercules, CA) and fixed by baking the membrane for 30 min at 80°C. After blocking the non-specific binding sites in blocking buffer (5% non-fat dry milk, 1% Tween 20 in 20 mM TBS, pH 7.6), the membrane was incubated with the antibody specific to CPDs for 1 h at room temperature. After washing, the membrane was incubated with HRP-conjugated secondary antibody. The circular bands of CPDs were detected by chemiluminescence using ECL detection system and autoradiography with HXR-photofilm (Hawkins Film, Oneonta, AL). The genomic DNA was used and tested from at least 3 independent set of experiments.

### RNA extraction and analyses of nucleotide excision repair (NER) genes using real-time polymerase chain reactions (RT-PCR)

The NHEK were subjected to UVB exposure with or without the treatment with silymarin. At desired time points, cells were harvested and cellular RNA was extracted using TRIzol reagents (Invitrogen, CA), as described [11], [14]. The mRNA expression of NER genes, such as *XPA, XPC, RPA1* and *DDB2*, was determined using real-time PCR. For the mRNA quantification, complementary DNA (cDNA) was synthesized using 3 µg RNA through a reverse transcription reaction (iScript™ cDNA Synthesis Kit, BIO-RAD, CA). Using SYBR Green/ Fluorescein PCR Master Mix (SuperArray Bioscience Corporation, MD), cDNA was amplified using real-time PCR with a BioRad MyiQ thermocycler and SYBR green detection system (BioRad, CA). Manufacturer-supplied (SuperArray, Bioscience Corporation, MD) primer pairs were used to measure the expression levels of NER genes. The standard PCR conditions were: 95°C for 15 min, then 40 cycles at 95°C, 30 sec; 55°C, 30 sec; and 72°C, 30 sec, as recommended by the manufacturer. Samples were run in triplicate to ensure amplification integrity. The expression levels of genes were normalized to the expression level of the β-actin mRNA in each sample, as performed earlier [11], [14]. For mRNA analysis the calculations for determining the relative level of gene expression were made using the cycle threshold (Ct) method. The threshold for positivity of real-time PCR was determined based on negative controls.

### UVB irradiation of mice

Mice were UVB-irradiated as described previously [9], [10]. Briefly, the clipper-shaved dorsal skin was exposed to UV radiation from a band of four FS20 UVB lamps (Daavlin, UVA/UVB Research Irradiation Unit, Bryan, OH) equipped with an electronic controller to regulate UV dosage. The UV lamps emit UVB (280–320 nm; ≈80% of total energy) and UVA (320–375 nm; ≈20% of total energy), with UVC emission being significantly less (<1%). The majority of the resulting wavelengths of UV radiation were in the UVB (290–320 nm) range with peak emission at 314 nm.

### Evaluation and detection of apoptotic or sunburn cells (SC) in mouse skin

NER-deficient mice and their wild-type counterparts (C3H/HeN) were exposed to UVB (240 mJ/cm^2^) radiation with or without pretreatment of the skin with silymarin (1 mg/cm^2^). The mice were sacrificed 24 h after UVB irradiation and skin samples were obtained, fixed in 10% formaldehyde and embedded in paraffin. Skin sections (5 µm thick) were stained with H&E following routinely used procedure. SCs were identified and counted throughout the epidermis in each section using light microscopy. The identification of apoptotic or SCs was based on morphologic characteristics, including cell membrane shrinkage and nuclear condensation attributable to fragmentation of the cells [16]. The SCs were counted throughout the epidermis of section per sample using 1 cm×1 cm grid inserted in a conventional microscope. SCs were counted on whole 1.0 cm long epidermal section. Data are presented in terms of number of sunburn cells/cm epidermal length section, n = 5/group. Student's *t*-test was used to test the significance of the differences.

### Statistical analysis

The statistical significance of difference between treatment and control groups was evaluated with one-way ANOVA followed by *post hoc* Dunn's test using GraphPad Prism version 4.00 for Windows, GraphPad Software, San Diego, California, USA, www.graphpad.com. A *P* value <0.05 was considered statistically significant.

## Results

### Silymarin protects NHEK from UV radiation-induced apoptosis

**Figure 1:**
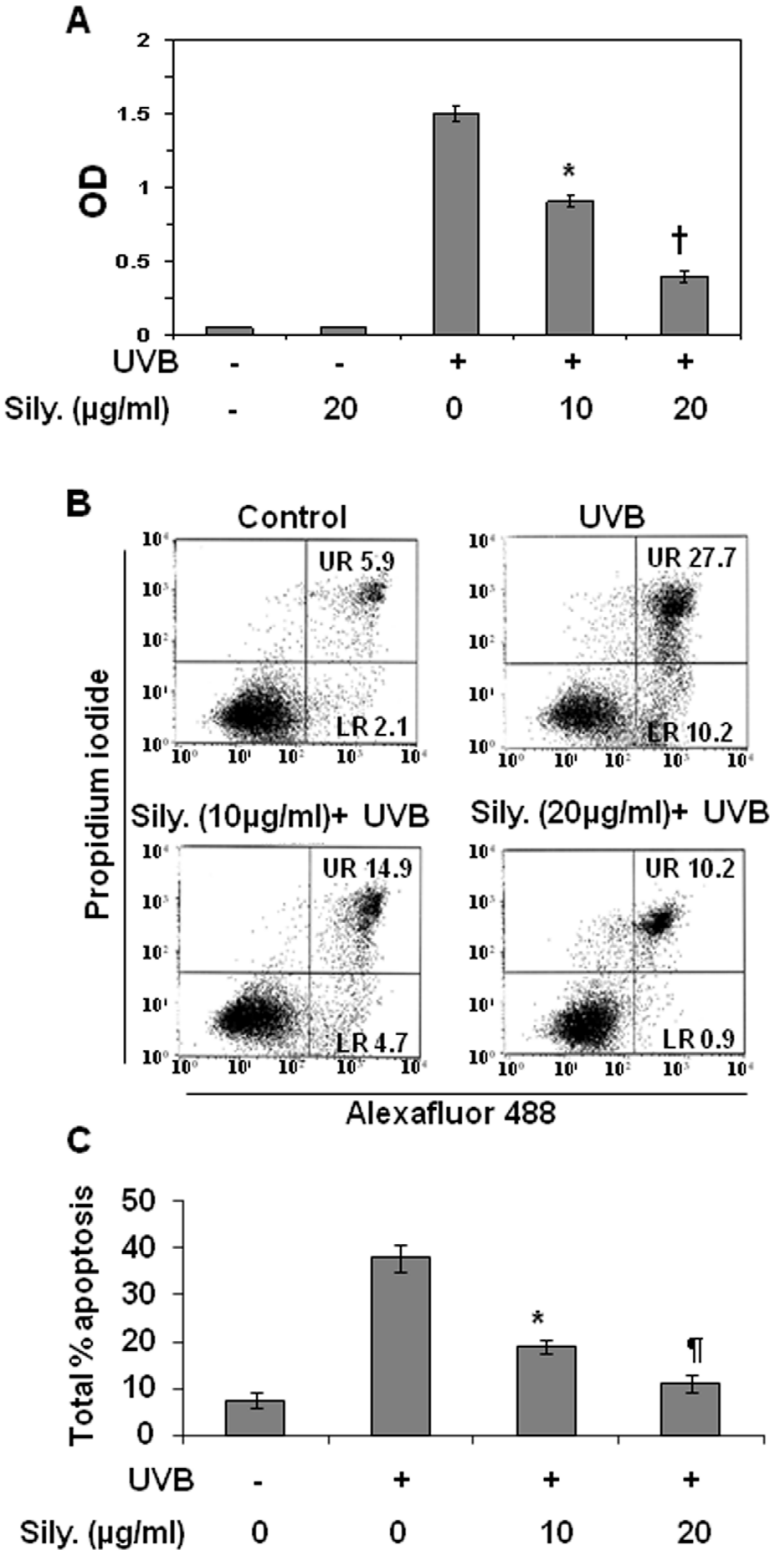
Treatment of NHEK with silymarin suppresses UVB-induced apoptotic cell death. **(A)**, Treatment of NHEK with silymarin inhibits UVB-induced cell death as determined by Cell Death Detection ELISA Kit following the manufacturer's protocol. Cells were treated with silymarin for 3 h before UVB irradiation. Cells were harvested 24 h after UVB exposure and subjected to the analysis of cell death. **(B)**, Silymarin inhibits UVB-induced apoptosis in NHEK. NHEK were exposed to UVB with and without the treatment with silymarin. Cells were harvested 24 hours later for the analysis of apoptotic cells by FACS using the Annexin V-Alexa Fluor488 Apoptosis Vybrant Assay Kit following the manufacturer's protocol. **(C)** Total percent of apoptotic cells (early+ late) in each treatment group was summarized and data are presented as mean±SD of three independent experiments. Sily. = silymarin. Statistically significant difference *vs* non-silymarin treated UVB exposed control, ^*^ *P*<0.01, ^¶^ *P*<0.005, ^†^ *P*<0.001.

To determine the protective effect of silymarin on UVB-induced apoptosis of NHEK, we treated NHEK cells with silymarin (0, 10 and 20 µg/mL) 3 h prior to UVB irradiation (150 J/m^2^). After UVB irradiation, cells were incubated with or without silymarin for additional 24 h followed by determination of DNA fragmentation using Cell Death ELISA kit. We found that exposure of cells with UVB resulted in significant induction of cell death compared with non-UVB-irradiated cells. Treatment of cells with silymarin in absence of UVB exposure did not elicit any effect on DNA fragmentation (Fig. 1A). However, treatment of cells with 10 µg/mL and 20 µg/mL of silymarin prior to and after UVB irradiation significantly protected the cells respectively by 40% (*P*<0.01) and 73% (*P*<0.001) from UVB radiation-induced apoptosis. To confirm the protective effect of silymarin on UVB radiation-induced apoptosis in NHEK, apoptosis was further assessed using the Annexin V-conjugated AlexaFluor 488 (Alexa488) Apoptotic Detection Kit, as previously described [15]. Apoptotic cells were counted as late or early apoptotic cells, which are shown respectively in the upper right and lower right quadrants of the histograms presented in Fig. 1B [17]. After 24 h of treatment, the silymarin-induced apoptosis of NHEK, in absence of UVB irradiation, was not significantly greater than that of vehicle-treated controls (data not shown). As shown in Fig. 1B, exposure of cells with UVB resulted in significant induction of apoptosis (37.9%, *P*<0.005) compared to non-UVB-exposed cells (8.0%). Treatment of NHEK with silymarin resulted in a significant reduction in the number of apoptotic cells at both the early and late stages of apoptosis. The total percentage of apoptotic cells in NHEK cells is summarized in Figure 1C. Our data indicated that treatment of cells with silymarin at the concentration of 10 µg/mL and 20 µg/mL significantly blocked UVB radiation-induced apoptosis and that is by 58% (*P*<0.01) and 84% (*P*<0.005) respectively.

### Silymarin reduces or repair DNA damage in UVB-exposed NHEK

**Figure 2:**
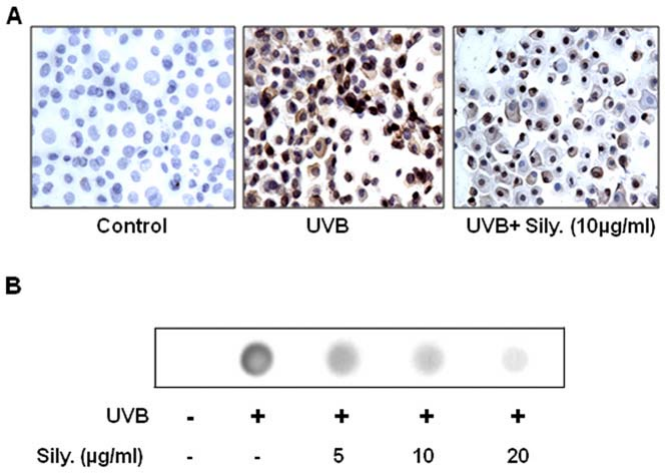
Silymarin stimulates DNA repair in UV-exposed NHEK. **(A),** NHEK were treated with silymarin for 3 h before UVB (150 J/m^2^) irradiation. Cells were harvested 36 h later, cytospun, and subjected to cytostaining to detect CPD^+^ cells, as detailed in Materials and methods. CPD-positive cells are dark brown. Magnification, x400. Photomicrographs are representative of three independent experiments. CPD+ cells were not detectable in non-UVB-exposed keratinocytes. **(B)** The analysis of damaged DNA in the form of CPDs was performed by dot-blot analysis using antibody specific to CPDs or thymine dimers. Genomic DNA from various treatment groups was subjected to dot-blot analysis using an antibody specific to CPDs. Results are shown from a single experiment and is representative of 3 independent experiments.

To determine whether silymarin reduces or repair UVB-induced DNA damage in NHEK, we have checked the effect of silymarin on UVB-induced DNA damage in the form of CPDs formation and their repair. For this purpose, cells were exposed to UVB (150 J/m^2^) with or without the treatment with silymarin. Cells were harvested either immediately or 36 h after UVB irradiation and subjected to the analysis of CPD-positive cells following cytostaining using CPD-specific antibody. CPD-positive cells were not detectable in non-UVB-irradiated cells whether or not they were treated with silymarin (Fig. 2A). When the cells were analyzed for CPDs immediately after UVB-exposure, no differences were observed in the cells treated with or without silymarin in terms of the number of CPD-positive cells (data not shown). This finding suggests that silymarin does not prevent immediate formation of CPDs after UVB exposure and excludes a UVB radiation filtering effect. When the cells were analyzed 36 h after UVB irradiation, the number of CPD-positive cells and intensity of staining of CPD-positive cells was markedly decreased in silymarin-treated cells compared to the cells which were not treated with silymarin but exposed to UVB (Fig. 2A), suggesting that silymarin might accelerate the repair of UVB-induced CPDs in NHEK.

The protective effect of silymarin on UVB-induced DNA damage was further verified using dot-blot analysis of genomic DNA isolated from the NHEK exposed to UVB with and without the treatment of silymarin (0, 5, 10 and 20 µg/mL). There was no significant difference in the dot-blot pattern of CPDs between cell samples obtained immediately after UVB exposure from UVB-exposed NHEK whether or not they were treated with silymarin (data not shown). In samples obtained 36 h after UVB exposure, the intensity of the dot-blot was markedly lower in the silymarin-treated NHEK in a dose-dependent manner than non-silymarin-treated UVB-exposed control cells. The genomic DNA sample obtained from the cells that were not exposed to UV was negative in the dot-blot assay, as shown in Figure 2B.

**Figure 3:**
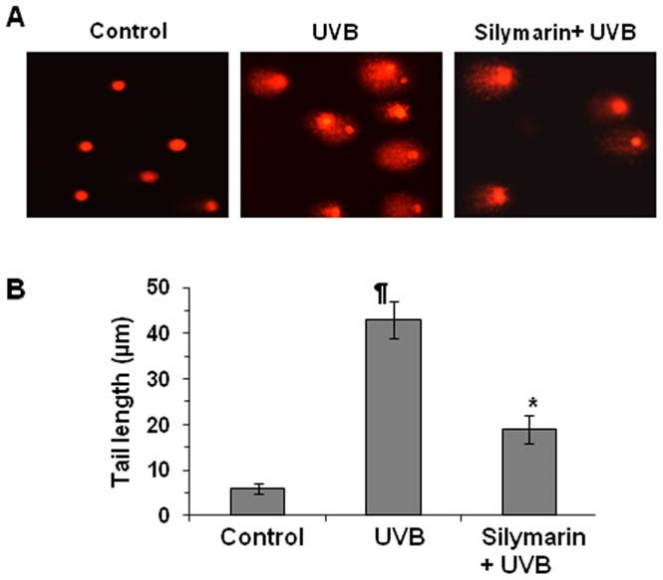
Silymarin prevents UVB-induced DNA damage as determined by comet assay. **(A),** NHEK were exposed to UVB (150 J/m^2^) radiation with and without the treatment with silymarin (20 µg/mL), as described under Fig. 2. Keratinocytes were harvested 36 h after UVB irradiation, and UVB-induced DNA damage was determined using comet assay, as detailed in Materials and methods. The comet assay was used to determine UVB-induced DNA damage in the form of DNA fragmentation. **(B),** The tail of the comet was measured in each cell under microscope and expressed in µm as a mean±SD from at least 30 cells in each treatment group. ^¶^Significant increase in tail length *versus* non-UVB-exposed control, p<0.001; ^*^Significant decrease in tail length versus UVB alone, p<0.001.

Finally, we also determined and verified photoprotective effect of silymarin on UVB-induced cellular DNA damage using comet assay, which was also used as a biomarker of apoptosis. As shown in Figure 3 (Panel A and B), exposure of NHEK with UVB radiation (200 J/m^2^) resulted in extensive DNA damage as reflected from the tail length of the comet compared to cells that were not exposed to UVB radiation. However, treatment of cells with silymarin (20 µg/mL) resulted in reduced amount of DNA damage or fragmentation compared to the cells which were not treated with silymarin but exposed to UVB, as is evident by the comet structure (Fig. 3A). DNA damaging effect and its prevention by silymarin in terms of DNA fragmentation was determined by measuring the tail length of the comet under microscope. The data of tail lengths in µm were represented as mean±SD from at least 30 cells or comets in each treatment group (Fig. 3B), which suggest that silymarin reduced UVB-induced DNA damage by 65% compared to UVB alone-exposed control cells.

### Silymarin enhances the levels of NER genes in UVB-exposed NHEK

**Figure 4:**
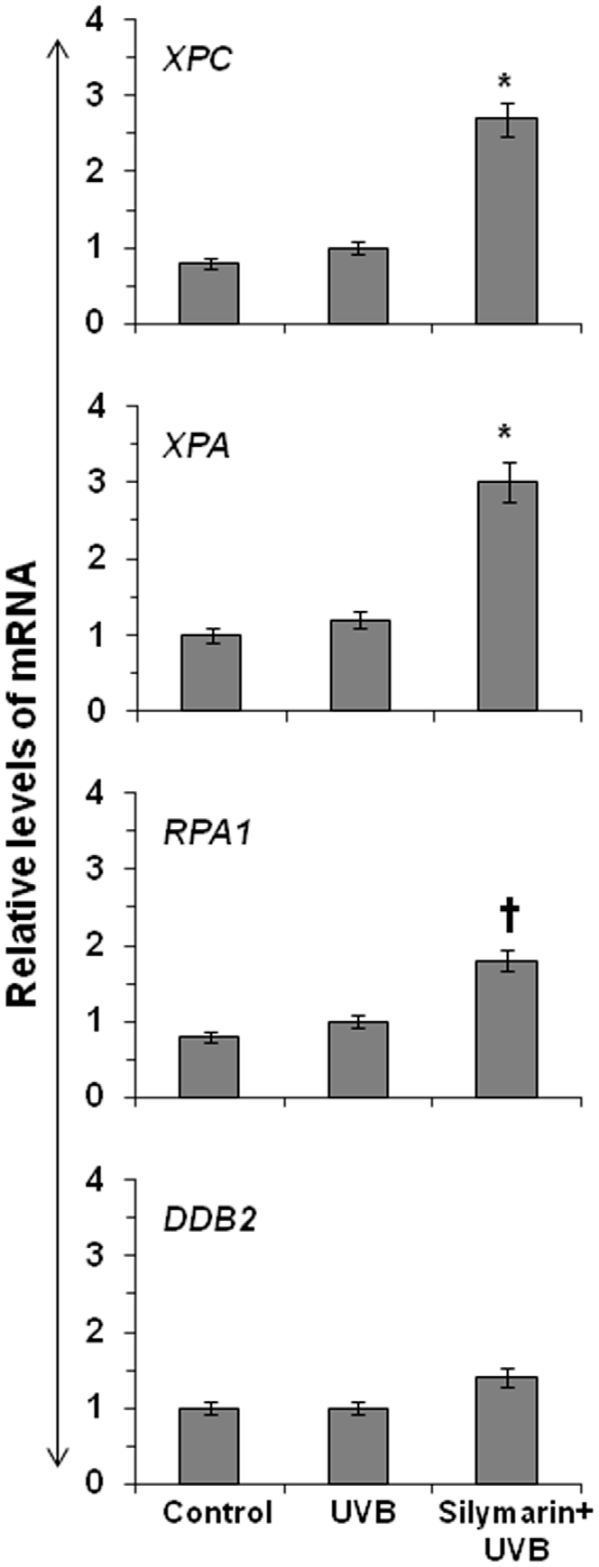
Silymarin stimulates the mRNA levels of NER genes in UVB-exposed NHEK. NHEK were exposed to UVB with and without the treatment of silymarin (20 µg/mL). Cells were harvested 1 h later and RNA was extracted. The mRNA levels of NER genes were determined using real-time PCR. The data of mRNA expression levels of various NER genes are expressed as mean±SD. Experiments were repeated three times. Statistically significant difference *versus* UVB alone, ^*^p<0.001, ^†^p<0.05.

As we have observed that silymarin enhances the repair of UVB-induced DNA damage in UVB-exposed NHEK, the next question was whether silymarin repairs DNA damage through the stimulation of NER genes? For this purpose, NHEK were exposed to UVB with and without the treatment of silymarin (20 µg/mL) in culture media 3 h before UVB exposure. Cells were harvested 1 h after UVB exposure, RNA isolated and subjected to the analysis of mRNA expression of some selected NER genes (*i.e.*, *XPA*, *XPC*, *RPA1*, and *DDB2*) using real-time PCR. The acute exposure of the NHEK with UVB slightly enhances the levels of NER genes (not significant) compared to non-UVB-exposed NHEK. As shown in Figure 4, the mRNA levels of NER genes were significantly enhanced (*P*<0.05 and *P*<0.001) in the UVB-exposed NHEK treated with silymarin compared to non-silymarin-treated UVB-exposed NHEK. Intriguingly, the enhancement of *XPA* and *XPC* gene levels after the treatment of cells with silymarin was significantly higher compared to the levels of other NER genes (*RPA1* and *DDB2*). Treatment of NHEK with silymarin alone for identical time period did not significantly induce the levels of NER genes (data not shown). These data suggest that silymarin might repair UVB-induced DNA damage in NHEK through the enhancement of the levels of *XPA* and *XPC* genes which have NER properties.

### Silymarin stimulates repair of UVB-induced DNA damage following NER mechanism

**Figure 5:**
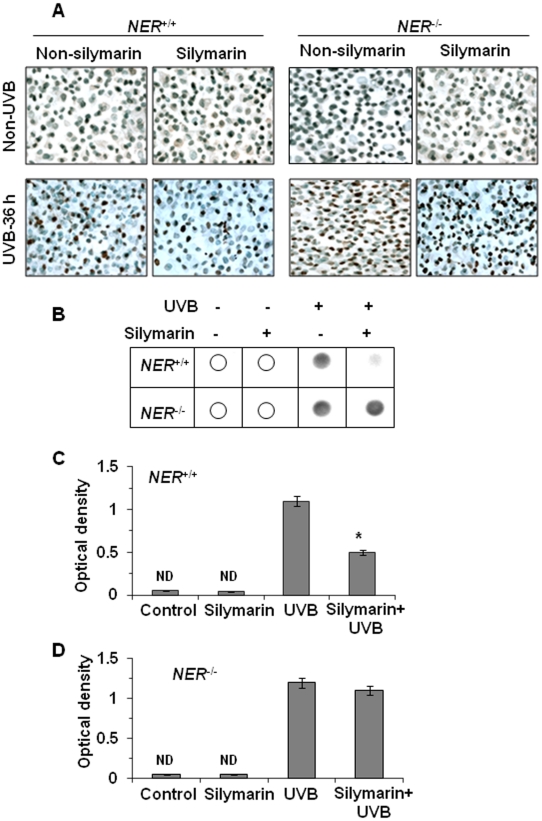
Silymarin protects UVB-induced DNA damage and cell death in NER-proficient fibroblasts but not in NER-deficient fibroblasts. **(A)**, NER-proficient and NER-deficient human fibroblasts were exposed to UVB (150 J/m^2^) with or without the treatment of silymarin (20 µg/mL) and cells were harvested 36 h later, cytospun, and subjected to cytostaining to detect CPD^+^ cells. CPD-positive cells are dark brown. CPD+ cells were not detectable in non-UVB-exposed cells. Magnification, x400. **(B),** The analysis of UVB-induced DNA damage in the form of CPDs was performed by dot-blot analysis. Genomic DNA from various treatment groups was subjected to dot-blot analysis using an antibody specific to CPDs. Results are shown from a single experiment and is representative of 3 independent experiments. **(C and D)**, UVB-induced cell death in NER-proficient and NER-deficient cells was detected by Cell Death Detection ELISA following manufacturer's protocol. Treatment protocol was same as reported in panels A and B. The amount of apoptotic cell death is reflected by increase of absorbance at 405 nm (optical density), as shown on the y-axis. ^*^ *P*<0.001. ND = not detectable.

As, it has been shown that *XPA* gene plays an indispensable role in the NER pathway [18], we further examined whether NER mechanism is required in silymarin-mediated DNA repair. For this purpose, NER-deficient fibroblasts from xeroderma pigmentosum complementation group A-patient and NER-proficient fibroblasts from healthy person were exposed to UVB with or without prior treatment with silymarin (20 µg/mL). Cells were harvested either immediately or 36 h after UVB irradiation and subjected to cytostaing using CPD-specific antibody. CPD-positive cells were not detectable in non-UVB irradiated cells whether or not they were treated with silymarin (Fig. 5A). When the cells were analyzed for CPDs immediately after UVB-exposure, no differences were observed in the NER-proficient or NER-deficient cells whether treated with or without silymarin in terms of the number of CPD-positive cells per microscopic field (data not shown). This observation suggests that silymarin does not prevent immediate formation of CPDs after UVB exposure and further excludes a possibility of UVB radiation filtering effect. When the cells were analyzed 36 h after UVB irradiation, the numbers of CPD-positive cells were significantly lowered (69%, p<0.001) in the NER-proficient cells (Fig. 5A) compared to non-silymarin-treated UVB-irradiated NER-proficient cells. In contrast, silymarin was not able to repair UVB-induced DNA damage in the form of CPDs in NER-deficient fibroblasts, suggesting that silymarin might accelerate the repair of UVB-induced CPDs through an NER mechanism.

To further verify our observations of silymarin in NER-deficient and NER-proficient system, we examined the effect of silymarin on UV-induced CPDs in NER-proficient and NER-deficient cells using southwestern dot blot analysis. For this purpose NER-deficient and NER-proficient human fibroblasts were exposed to UV radiation in the presence or absence of silymarin, as described above. Cells were harvested 36 h later, genomic DNA was isolated and subjected to dot-blot analysis. As clearly indicated in Figure 5B, silymarin treatment of NER-proficient cells for 36 h resulted in remarkable repair or reduction in the levels of UV-induced CPDs compared with non-silymarin-treated UVB-exposed NER-proficient (NER^+/+^) cells, which was evident by the less intense dot-blot. However, this DNA-repairing effect of silymarin was not evident in the NER-deficient cells 36 h after UV irradiation. This may be due to absence of NER genes in these cells. The cells whether NER-deficient or NER-proficient and either treated with silymarin or not treated with silymarin did not show the presence of CPDs as reflected from the absence of dot blot (Fig. 5B).

### Silymarin protects cells from UVB-induced apoptotic cells death following NER mechanism

Further, we examined whether silymarin protects cells from UVB-induced apoptotic cell death following NER mechanism. For this purpose NER-proficient and NER-deficient human fibroblasts were exposed to UVB (150 J/m^2^) radiation with and without the treatment of silymarin following the same protocol as described above. After UVB irradiation, cells were further incubated with or without silymarin for additional 36 h followed by determination of DNA fragmentation using Cell Death ELISA kit. We found that exposure of NER-proficient cells with UVB resulted in significant induction of cell death compared with non-UVB-irradiated control cells. However, treatment of NER-proficient cells with silymarin prior to and after UVB irradiation significantly (*P*<0.001) protected the cells from UVB radiation-induced apoptosis (Fig. 5C). In contrast, treatment of NER-deficient cells with silymarin prior to and 36 h after UVB irradiation could not significantly protect the cells from UVB radiation induced cell death or apoptosis (Fig. 5D). Treatment of NER-proficient or NER-deficient cells with and without silymarin in absence of UVB exposure did not elicit significant effect on DNA fragmentation or cell death, and largely it was undetectable (Fig. 5C and 5D).

### Silymarin reduces the number of sunburn cells in UVB-exposed NER-proficient mice but not in NER-deficient mice

**Figure 6:**
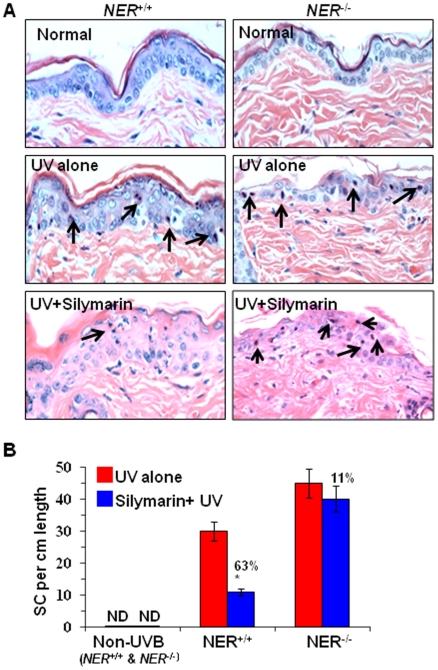
Effect of silymarin on UVB-induced sunburn cells in the skin of NER-deficient and their wild-types. (A), Silymarin repairs UVB-induced sunburn cells in NER-proficient mouse skin but not in NER-deficient mouse skin. Mice were exposed to UVB (240 mJ/cm^2^) with or without the treatment of silymarin, and sacrificed 24 h later. Skin samples were collected and subjected to H&E staining for the analysis of sunburn cells under microscope. Sunburn cells are shown by dark brown, n = 5/group. Some sunburn cells are shown by arrows. (B), The number of sunburn cells were counted per 1 cm length of epidermis from each mouse, and data are summarized in terms of mean±SD, n = 5 mice/group. Significant less number of SCs in NER^+/+^ mouse skin *vs* non-silymarin-treated NER^+/+^ wild-type mice, ^*^ *P*<0.001. ND = not detectable.

Next, we examined the effect of silymarin on UVB-induced sunburn cell formation using NER-proficient and NER-deficient mouse model. UVB-induced sunburn cells are considered as apoptotic cells in the skin. For this purpose, NER-deficient and their wild-type (NER-proficient) mice were used. NER-deficient mice are sensitive to UVB-induced DNA damage and photocarcinogenesis because they are deficient in DNA repair mechanism. In contrast, the wild-type mice are relatively resistant to UVB-induced DNA damage and photocarcinogenesis because of presence of NER system. Keeping this difference in mind, NER-deficient mice were exposed to 50 mJ/cm^2^UVB dose while wild-type mice were exposed to 240 mJ/cm^2^ UVB dose with and without topical treatment with silymarin (1 mg/cm^2^ skin area). Using these UVB doses we were able to induce approximately equivalent number of sunburn cell formation in two different mouse strains. Although, the UV irradiation doses are unequal, we can compare the chemopreventive effects of silymarin on sunburn cells on both strains of mice separately. Mice were sacrificed 24 h after UVB irradiation. Skin samples were collected, kept in 10% formalin buffer, and paraffin blocks were obtained. Microscopic evaluation of H&E stained skin sections revealed that the number of sunburn cells were higher in UVB-exposed NER-deficient and their wild-type mouse skin compared with non-UVB-exposed skin of these strains of mice. Sunburn cells are shown by dark brown. Treatment of NER-proficient mouse skin with silymarin resulted in significant reduction in number of sunburn cells (63%, *P*<0.001) compared with non-silymarin treated UVB-exposed mouse skin (Figures 6A and 6B). In contrast, we could not find a significant difference in the number of sunburn cells in the silymarin-treated or non-silymarin-treated UVB exposed NER-deficient mouse skin (Figures 6A, 6B). The numbers of sunburn cells in each treatment group are summarized in Figure 6B in terms of mean±SD in 1 cm length of the epidermis (n = 5/group). Sunburn cells were not detectable in the non-UVB-exposed mouse skin, whether it is treated with silymarin or not treated with silymarin. These data suggest that repair of UVB-induced sunburn cells by silymarin required active NER system.

## Discussion

In the present study, we demonstrate a novel mechanism by which silymarin, a plant flavanoid, prevents UVB-induced apoptosis and enhances DNA repair in UVB-exposed skin cells. UVB radiation-induced apoptosis has been extensively studied in human keratinocytes, which is the major cellular target for solar UVB radiation. UVB-induced apoptosis has been recognized as a protective mechanism because it contributes to the removal of cells carrying DNA damage, thereby preventing malignant transformation [2]. Cells undergo apoptosis because of irreparable DNA damage. If this damage can be repaired, cells may avoid apoptosis as well as cells may avoid abnormal deregulation, proliferation or replication of damaged DNA containing cells, and thus malignancy can be inhibited. Our data demonstrate that silymarin prevents UVB-induced apoptosis in skin cells, and this prevention is due to repair of damaged DNA caused by exposure of the cells to UVB radiation.

We show that silymarin stimulates repair of UVB-induced DNA damage and that leads to the prevention of apoptosis in UVB-exposed human epidermal keratinocytes as well as fibroblasts. UVB radiation induces DNA damage either through DNA fragmentation and/or the formation of CPDs in cells. In addition, UVA in particular is also responsible for oxidative DNA damage, and that can be repaired by base excision repair mechanism. As UVB-induced CPDs have been recognized as a molecular trigger for the induction of immunosuppression as well as an initiator of skin carcinogenesis, we focused our attention on the repair mechanism of DNA damage by silymarin. Silymarin repairs UVB-induced DNA fragmentation as demonstrated by comet assay as well as the formation of CPDs in UVB-exposed cells, and this may be one of the possible mechanisms by which silymarin inhibits UVB radiation-induced skin tumor development in mice. As, silymarin has been shown to have anti-oxidant effect in UVB-exposed mouse skin, the repair of UVB-induced damaged DNA may also be mediated, at least in part, through antioxidant effect of silymarin [8], [9].

NER is a major mechanism of DNA repair in mammalian cells. Since the treatment of cells with silymarin enhances the repair of UVB-induced DNA damage, we further examined whether the repair of UV-induced CPDs by silymarin is mediated via induction of NER genes. Our real-time PCR data reveal that treatment of NHEK with silymarin enhances the levels of NER genes (*e.g.*, *XPA*, *XPC, RPA1 and DDB2*) in UVB-exposed NHEK compared to non-silymarin-treated cells and that may have contributed in the rapid repair of damaged DNA in NHEK. The role of NER mechanism was further confirmed by assessing the effect of silymarin on UVB-induced DNA damage in human NER-deficient (or *XPA*-deficient) cells obtained from human patients suffering from xeroderma pigmentosum disease and NER-proficient cells were obtained from normal healthy person. Cells derived from patients suffering from xeroderma pigmentosum either lacks or have reduced DNA repair capacity due to genetic mutations in several components of the *NER*. The *XPA* complementation type represents the most severe phenotype, because the *XPA* gene is the most crucial component in the DNA repair process and, thus, cells lacking the *XPA* gene are completely deficient in NER [16]. Our immunostaining and dot-blot data indicated that silymarin was able to repair UV-induced CPDs in NER-proficient cells but was not able to repair in NER-deficient cells. These observations support the evidence that repair of UV-induced DNA damage in skin cells by silymarin is mediated through the NER-dependent mechanism. XPA is part of the core incision complex of the NER system [19]. Therefore, *XPA*-deficient mice are severely deficient in NER [12]. Because UV-induced DNA damage is known to be the major molecular trigger for the induction of apoptosis [3], NER-deficient mice have a higher number of apoptotic keratinocytes than wild-type counterparts, and therefore have a higher risk of developing UV-induced skin cancer upon chronic UV exposure due to the impaired capacity to remove UV-induced DNA damage [12]. *XPA*-deficient mice are also more susceptible to UV-induced immunosuppression as lower UV doses are required to achieve the same level of immunosuppression as in wild-type mice [20]. Following the repair of UVB-induced DNA damage, silymarin was able to inhibit UVB-induced apoptosis in NER-proficient cells, however this effect was not found in NER-deficient cells. Similar observations related with the role of *XPA* in DNA repair in UVB-exposed skin cells were also found by the treatment of α-melanocyte-stimulating hormone [21]. Administration of green tea polyphenols in drinking water of mice have been shown to prevent photocarcinogenesis in mice through enhanced repair of damaged DNA in UVB-exposed skin, and DNA repairing process has been shown to be mediated through stimulation of interleukin-12 in mice [11], [22]. Further, we present additional evidences using NER-deficient mouse model. Using this genetically modified mouse model, we found that silymarin does not prevent UVB-induced sunburn cell formation in NER-deficient mice but prevents in their wild-type counterparts. These findings have important implications for the chemoprevention of skin cancer by silymarin, and identify a new mechanism by which silymarin enhances DNA repair in UVB-exposed skin and that may have contributed in prevention of UV-induced skin tumor development.
